# Nanoparticles: A Hope for the Treatment of Inflammation in CNS

**DOI:** 10.3389/fphar.2021.683935

**Published:** 2021-05-26

**Authors:** Feng-Dan Zhu, Yu-Jiao Hu, Lu Yu, Xiao-Gang Zhou, Jian-Ming Wu, Yong Tang, Da-Lian Qin, Qing-Ze Fan, An-Guo Wu

**Affiliations:** ^1^ Sichuan Key Medical Laboratory of New Drug Discovery and Drugability Evaluation, Luzhou Key Laboratory of Activity Screening and Druggability Evaluation for Chinese Materia Medica, Key Laboratory of Medical Electrophysiology of Ministry of Education, School of Pharmacy, Southwest Medical University, Luzhou, China; ^2^ Department of Anesthesia, Southwest Medical University, Luzhou, China; ^3^ Department of Pharmacy, Affiliated Hospital of Southwest Medical University, Luzhou, China

**Keywords:** neurodegenerative diseases, central neural system, blood-brain barrier, neuroinflammation, nanoparticles

## Abstract

Neuroinflammation, an inflammatory response within the central nervous system (CNS), is a main hallmark of common neurodegenerative diseases, including Alzheimer’s disease (AD), Parkinson’s disease (PD), and amyotrophic lateral sclerosis (ALS), among others. The over-activated microglia release pro-inflammatory cytokines, which induces neuronal death and accelerates neurodegeneration. Therefore, inhibition of microglia over-activation and microglia-mediated neuroinflammation has been a promising strategy for the treatment of neurodegenerative diseases. Many drugs have shown promising therapeutic effects on microglia and inflammation. However, the blood–brain barrier (BBB)—a natural barrier preventing brain tissue from contact with harmful plasma components—seriously hinders drug delivery to the microglial cells in CNS. As an emerging useful therapeutic tool in CNS-related diseases, nanoparticles (NPs) have been widely applied in biomedical fields for use in diagnosis, biosensing and drug delivery. Recently, many NPs have been reported to be useful vehicles for anti-inflammatory drugs across the BBB to inhibit the over-activation of microglia and neuroinflammation. Therefore, NPs with good biodegradability and biocompatibility have the potential to be developed as an effective and minimally invasive carrier to help other drugs cross the BBB or as a therapeutic agent for the treatment of neuroinflammation-mediated neurodegenerative diseases. In this review, we summarized various nanoparticles applied in CNS, and their mechanisms and effects in the modulation of inflammation responses in neurodegenerative diseases, providing insights and suggestions for the use of NPs in the treatment of neuroinflammation-related neurodegenerative diseases.

## Introduction

Neuroinflammation is characterized by the activation of microglia and astrocytes, as well as the release of cytokines and reactive oxygen species. It may cause synaptic dysfunction, the loss of synapses, and neuron damage. Since neuroinflammation is the common mechanism behind various CNS-related diseases, alleviation and inhibition of neuroinflammation has become a research hotspot over recent years. However, most drugs with anti-inflammatory characteristics cannot cross the blood–brain barrier to the target cells such as microglia and astrocytes. The BBB is formed by the brain capillary wall, glial cells and the barrier between plasma and cerebrospinal fluid (CSF) that is formed by the choroid plexus. The BBB is an essential defense mechanism of the CNS that restricts the transit of toxins or pathogens and selectively allows individual molecules to pass. However, the BBB also significantly hinders drug delivery to the CNS (Zhou et al., 2018; Tosi et al., 2020).

Nanomaterials can make dramatic changes to the treatment of neuroinflammation. Over recent decades, a rising number of nanomaterials have been developed. There is increasing optimism that nanomedicine will continue to develop and could even change the model of the prevention, diagnosis and treatment of disease (Rodallec et al., 2018; Avasthi et al., 2020). Nanomaterials are made up of engineered materials or devices with the smallest functional organizations in the size range of 1–100 nm (Zielinska et al., 2020). They are mainly classified into two groups: inorganic and organic nanomaterials. Inorganic nanomaterials come in an array of forms, including Au nanoparticles, TiO_2_ NPs, IONPs and other metal NPs. Organic nanomaterials mainly include lipid NPs (liposomes and solid lipid NPs), nanoemulsions and polymer NPs (polymeric NPs, dendrimers, nanogels, and micelles) (Kumari et al., 2010; Martinez-Lopez et al., 2020).

NPs can encapsulate drugs with relatively high drug loading (Sim et al., 2020), and the surface of NPs can be easily manipulated to achieve drug targeting (Sun et al., 2014). In addition, NPs can control the release of drugs at the site of target cells or tissues, thereby increasing therapeutic efficacy and reducing the side effects of drugs. Drugs that are insoluble or unstable in aqueous phase could be formulated into nano delivery systems, which improves their solubility and extends their pharmacologic effects. Most importantly, NPs systems could provide a variety of choices for the routes of drug administration, including intravenous, nasal, oral, parenteral, intra-ocular, and dermal topical application (Spuch et al., 2012; Carita et al., 2018; He et al., 2019; Islam et al., 2020). In recent years, a number of NPs have been developed as effective and minimally invasive carriers to help other drugs cross the BBB or as the therapeutic agents for the treatment of neuroinflammation-mediated neurodegenerative diseases (Moura et al., 2019; Tang et al., 2019; Tosi et al., 2020).

In this article, we summarize the current knowledge gained from recent advances in nanomaterials, and their key treatment roles in neuroinflammation-related neurodegenerative diseases, which provides more opportunities and prospects for the therapy of neurodegenerative diseases in the future.

## Neuroinflammation in CNS-Related Diseases

Neurodegenerative diseases are the main type of CNS-related diseases and include Alzheimer’s disease, Parkinson’s disease, Huntington disease (HD), frontotemporal dementia (FTD), Lewy body dementia (LBD), etc. The pathologies of neurodegenerative diseases are characterized by neuroinflammation, cerebral protein aggregates, synaptic abnormalities, and progressive loss of neurons (Dugger and Dickson, 2017; Vaquer-Alicea and Diamond, 2019). Gradual cognitive and memory impairments and disorder in movements are common clinical symptoms (Katsnelson et al., 2016; Stephenson et al., 2018).

**FIGURE 1:**
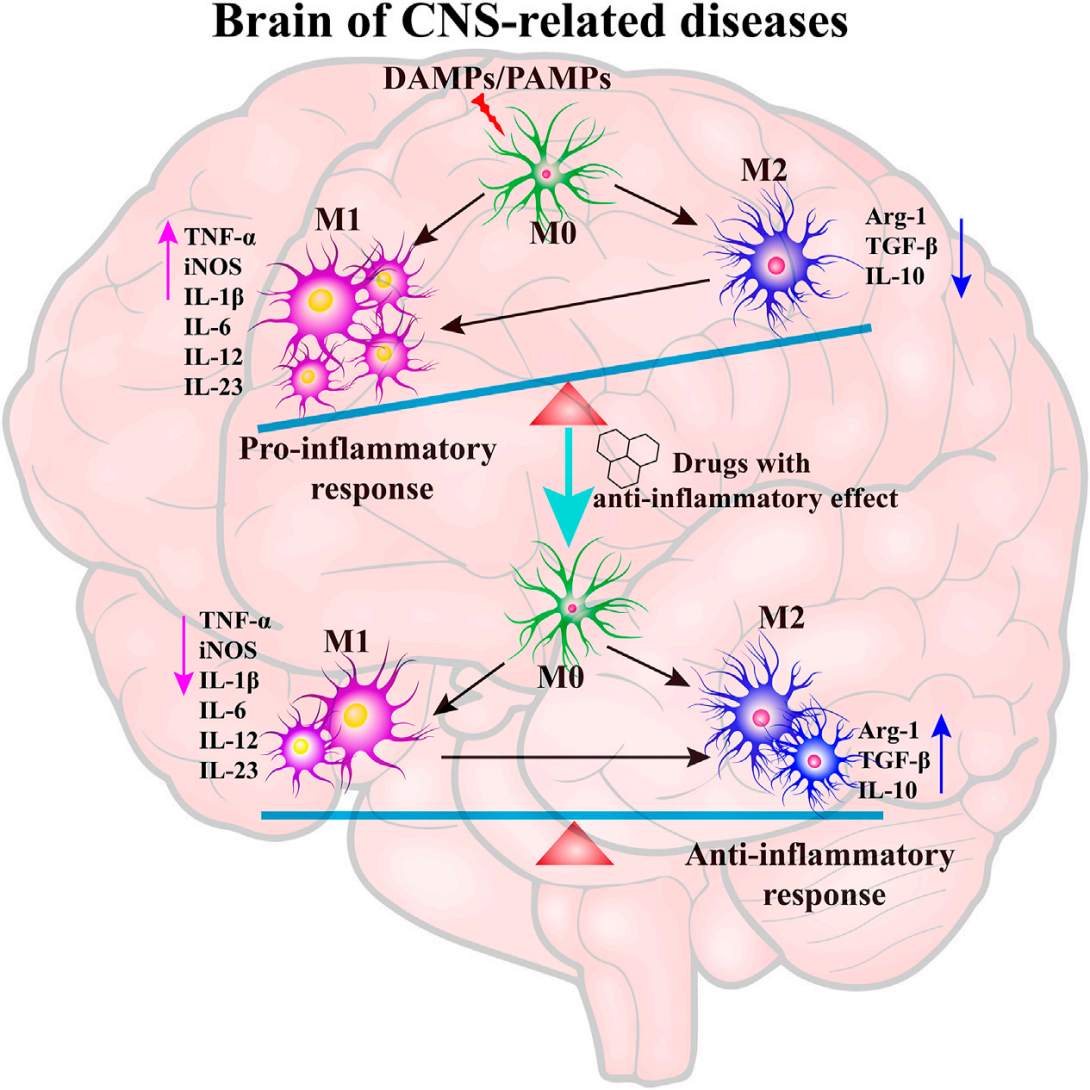
The key role of neuroinflammation in neurodegenerative diseases. The resting microglia (M0) are over-activated by PAMPs/DAMPs into a pro-inflammatory state (M1), which leads to the generation of pro-inflammatory cytokines. The treatment of anti-inflammatory drugs can inhibit the over-activation of microglia and promote the microglia into an anti-inflammatory state to maintain the balance of M1/M2 type microglia.

Neuroinflammation generally refers to an inflammatory response within the CNS or activation of the neuroimmune cells, microglia and astrocytes into the state of pro-inflammatory response (Schain and Kreisl, 2017). Emerging evidence indicates that the resting microglia (M0) is over-activated by various pathogen-associated molecular patterns (PAMPs) or danger-associated molecular patterns (DAMPs) including particulates, viruses, bacteria, fungi, toxins, lipopolysaccharide (LPS), crystals, silica, and misfolded protein aggregations (Aβ, Tau, α-synuclein, etc.) in neurodegenerative diseases (Agostinho et al., 2010; Allaman et al., 2011; Alcendor et al., 2012; Niranjan, 2014). The transient receptor potential melastatin-related 2 (TRPM2) is a calcium-permeable channel induced by oxidative stress (Alawieyah Syed Mortadza et al., 2018), ultimately causing activation of the NLRP3 inflammasome (Koenigsknecht and Landreth, 2004). Microglia and astrocytes are the primary constituents of a dedicated neuroimmune system in CNS. The moderative activation of microglia (M2) can protect brain by defending against harmful materials by releasing many anti-inflammatory cytokines, including Arg-1, TGF-β, and IL-10. However, amplified, exaggerated, or chronically activated microglia (M1) lead to robust pathological changes and neurobehavioral complications, such as depression and cognitive deficits (Norden and Godbout, 2013). The inflammation process is indicated by the production of pro-inflammatory cytokines, including IL-1β, IL-6, IL-18 and tumor necrosis factor-α (TNF-α), as well as many chemokines, such as C-C motif chemokine ligand 1 (CCL1), CCL5, and C-X-C motif chemokine ligand 1 (CXCL1), and small-molecule messengers, including prostaglandins and nitric oxide (NO), and reactive oxygen species (DiSabato et al., 2016). After treatment with anti-inflammatory drugs, the M1-type microglia are converted into M2-type microglia, which is indicated by the decrease of pro-inflammatory cytokines and increase of anti-inflammatory cytokines (Figure 1).

Many researchers have recently reported findings about the mechanism of neuroinflammation associated with neurodegenerative disorders (Schain and Kreisl, 2017). Earlier studies identified amyloid β (Aβ) and hyperphosphorylated tau as playing essential roles in the progress of AD (Eftekharzadeh et al., 2018; Nakamura et al., 2018). Many previous studies found that Aβ oligomers are the most toxic forms among all Aβ species, and the smaller oligomers of Aβ have been proved to be stronger stimuli to activate the microglial cells (Yang et al., 2017). The aggregated tau has been considered to induce microglial changes by activating the NLRP3–ASC axis (Ising et al., 2019; Stancu et al., 2019). Numerous studies have shown that Aβ and hyperphosphorylated tau induce pro-inflammatory conditions *in vitro* and vivo (Maezawa et al., 2011; Morales et al., 2013; Asai et al., 2014; Marlatt et al., 2014; Shi et al., 2019). Moreover, accumulating evidence suggests that soluble α-synuclein aggregates play a significant role in PD and most of them were found within the substantia nigra pars compacta (SNc) region of the midbrain (Winner et al., 2011; Choi et al., 2013). Recently, activated microglia were found surrounding Lewy bodies, suggesting that neuroinflammation is a common response to α-synuclein aggregates (Streit and Xue, 2016). In addition, widespread microglial activation was visible by positron emission tomography (PET) in the brain of living ALS patients and SOD1^G93A^ mice, indicating that there is an association between neuroinflammation and ALS (Turner et al., 2004; Corcia et al., 2012; Gargiulo et al., 2016). Therefore, microglia over-activation and the resulting neuroinflammation have been implicated in neurodegenerative diseases, while the inhibition of neuroinflammation has been considered a promising strategy for the treatment of neuroinflammation-mediated neurodegenerative diseases.

## The Effect of NPs in CNS-Related Diseases

**FIGURE 2:**
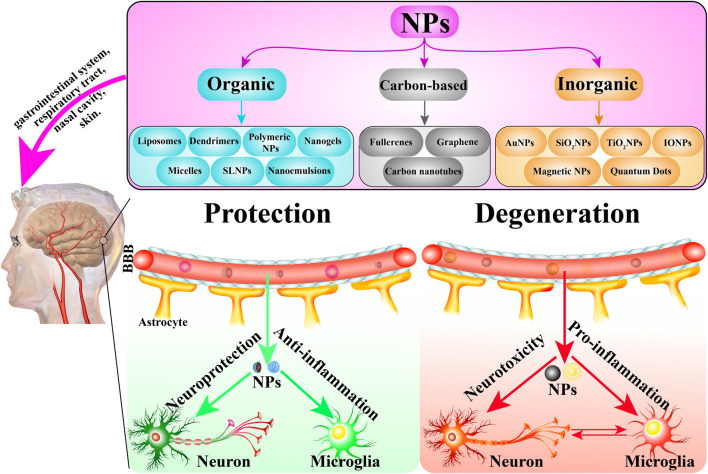
The classification of NPs and the role of NPs in CNS-related diseases. NPs are mainly classified into three groups: organic, carbon-based, and inorganic NPs. In general, these NPs are administrated via the gastrointestinal system, respiratory tract, nasal cavity, and skin, etc. They cross the BBB into the target brain cells including neurons, microglia and astrocyte to exert protective and degenerative effects.

The traditional definition of nanoparticle size is 1–100 nm. Indeed, while most NPs are under 100 nm, the diameter of some composite or drug-loaded NPs are over 100 nm (Suk et al., 2016; Tosi et al., 2020). Furthermore, the generally accepted classification of nanoparticles is based on their organic, inorganic, and carbon-based nature (Figure 2). Particle size is the basic attribute of NPs, which determines the biological fate, toxicity, distribution, and targeting ability of NPs to a certain extent. Generally, smaller NPs are prone to aggregate during dispersion, storage, and transport, and exhibit faster drug release due to their larger surface-to-volume ratio. On the contrary, larger NPs lead to faster polymer degradation and slower drug release (Gupta et al., 2019; Zahin et al., 2020). The shape of NPs contributes to biological functions such as drug delivery, half-life period, endothelial intake, and targeting ability (Petros and DeSimone, 2010; Yoo et al., 2010; Zhang et al., 2015). NPs have varied shapes including rod, spherical, triangular, cube, hexagonal, fivefold star shape and monodisperse cubic dendrites, among others (Lacroix et al., 2012; Sun et al., 2014). Surface charge and hydrophobicity are surface properties of NPs, which may influence their biodistribution, circulation time, and toxicity (Arvizo et al., 2011). Positively charged NPs show better efficacy of imaging, gene transfer, and drug delivery, but they are reported to possess higher cytotoxicity. Hydrophobicity is another important surface property, which plays an important role in plasma protein binding and clearance via the reticuloendothelial system (RES) (Frohlich, 2012; Nam et al., 2013).

To date, NPs have been widely used in CNS-related diseases including neurodegenerative disease, traumatic brain injury, stroke, and cerebral tumor. As drug carriers or as therapeutic drugs by themselves, NPs show potential for neuroprotective effects by oxidation resistance, anti-apoptosis, and nerve regeneration (Figure 2). The initial focus of neuroprotective treatment is the neurons, which are considered the most vulnerable cells to hypoxia and excitotoxicity. However, in recent years, concerns have been extended to astrocytes, pericytes, endothelial cells, and other neural cells, targeting antioxidant enzymes, antiapoptotic pathways, and downstream cytokines (Moretti et al., 2015; Chamorro et al., 2021). Polysorbate 80 (PS80) reduced the secondary spread of neuroinflammation and injury in traumatic brain injuries (TBI) by preventing the spread of reactive oxygen species (ROS) (Yoo et al., 2017a). Poly (lactic-co-glycolic acid) nanoparticles, which encapsulated Lexiscan and Nogo-66, improved stroke survival, suggesting the potential therapeutic effect for stroke (Han et al., 2016). Numerous researchers have demonstrated that organic and inorganic NPs might be helpful in the treatment of neurodegenerative diseases, especially AD and PD. The possible mechanisms include the delivery of a corresponding drug, siRNA transfection, interference with Aβ fibril formation, down-regulating proinflammatory factors, etc. (Tiwari et al., 2014; Karthivashan et al., 2018; Baskin et al., 2020).

NPs can participate in the treatment of neuroinflammation as carriers for therapeutic drugs including curcumin, okadaic acid, quercetin, anthocyanin, and levodopa. With the assistance of NPs, the drugs can cross the BBB to target cells more easily, thereby inhibiting inflammatory pathways and the release of inflammatory cytokines. Besides, magnetic NPs, such as IONPs, have been applied in diagnosis and imaging. Moreover, nanoparticles themselves also have therapeutic effects in neuroinflammation. For example, AuNPs could induce microglia polarization toward the M2 phenotype (Xiao et al., 2020), carbon nanotubes (CNTs) can integrate with neurons and enhance neuronal functions (Matsumoto et al., 2007), and rhubaric acid hydrogel inhibits TLRs signaling pathways (Zheng et al., 2019).

Although NPs exhibit potent neuroprotection and anti-inflammatory effects, many NPs have been reported to exhibit neurotoxicity and pro-inflammatory responses in some cells and animals with CNS-related diseases (Table 1; Figure 2). For example, copper NPs can cause BBB dysfunction, swelling of astrocytes, and neuronal degeneration once introduced into the bloodstream (Sharma, 2009; Sharma et al., 2009).

**TABLE 1:** The role of nanoparticles in neurotoxicity and pro-neuroinflammation.

NPs	Diameter (nm)	Cells/animals	Treatment time	Administration route	Dose	Mechanism and detected markers	References
***In vitro***							
MWCNTs	5–15 nm	3D brain organoids derived from iPSCs	24 h		0 μg/ml, 16 μg/ml, and 64 μg/ml	NF-κB-KLF4 pathway; nNOS	Jiang et al. (2020)
ZnO NPs	19.61 ± 5.83 nm	PC12 cells	6 h or 12 h		0–20 μg/ml	CAMK2A/CAMK2B pathway Oxidative stress: GSH, MDA, NO, SOD Inflammatory cytokines: IL-1β, TNF-α	Liu et al. (2020a)
IONP, IONP-TPP and IONP-APM	11 nm	Rotenone-induced SH-SY5Y cells	24 h or 48 h		0–200 μg/ml	AMPK pathway	Huang et al. (2019)
Silica NPs	50, 100, and 300 nm	N9, bEnd.3, and BV-2 cells	24 h		25–200 μg/ml	Oxidative stress: ROS, LDH Pyroptosis: N-GSDMD Inflammatory cytokines: IL-1β	Du et al. (2019)
Mn_3_O_4_ NPs	18.98 ± 4.61 nm	PC12 cells	24 h		5 μg/ml, 10 μg/ml, and 20 μg/ml	Oxidative stress: ROS, Ca^2+^, LDH Apoptosis: Bax/Bcl-2, caspase-3, caspase-9	Chen et al. (2020)
Co. NPs	Under 100 nm	SH-SY5Y cells	24 h at day 4 and day 12		1–100 µg/ml	Oxytosis:ROS, Ca^2+^, GSH, GPX4	Gupta et al. (2020)
Ag NPs	20 and 70 nm	Pure cortical neurons from SD rat embryos on embryonic day 18	24 h		0.01–40 µg/ml	Extracellular dopamine, cytoskeleton changes	Zhang et al. (2020)
***In vivo***							
ZnO NPs	42.31 ± 17.94 nm	Male Wistar rats	30 days	Tongue instillation	134.2 mg/kg and 536.8 mg/kg	NF-κB and MAPK pathways Inflammatory cytokines: TNF-α, IL-1β, IL-6, IL-10, IFNG, NOS2	Liang et al. (2018)
Al_2_O_3_ NPs	22.63 ± 5.64 nm	Male Wistar rats	15–30 days	Tongue instillation	20 μg/g	Oxidative stress: MDA Inflammatory cytokines: TNF-α, IL-1β	Liu et al. (2020b)
CeO_2_-NPs	Under 50 nm	Oncorhynchus mykiss juveniles	28 days	Aquarium’s exposure	0.1 μg/L, 0.01 μg/L, and 0.001 μg/L	Oxidative stress: GSTs and catalase	Correia et al. (2019)
f-CNTs	20–30 nm	Female C57/Bl6 mice	Single injection	Stereotactic administration	500 ng/mouse	Inflammatory cytokines: IL-10, TNF-α, and IL-1β	Bardi et al. (2013)

## Organic NPs

### Lipid-based NPs

#### Liposomes

Liposomes are vesicular drug-delivery systems containing an aqueous inner core enclosed in multi-lamellar phospholipid bilayers. Hydrophobic and hydrophilic drugs can be loaded in the phospholipid bilayers and aqueous core, respectively (Agrawal et al., 2017; Li et al., 2018).

Liposomes have the characteristics of nanoscale, ideal biocompatibility and relative stability. Due to the structural similarity of phospholipid bilayers to the cell membrane, liposomes can be absorbed by vascular endothelial cells more easily, which makes them promising drug-delivery systems to increase the BBB crossing of therapeutics in CNS diseases associated with neuroinflammation (Li et al., 2017; Patel and Patel, 2017; Li et al., 2019a). However, they can easily be degraded and scavenged by macrophages, and their binding to plasma proteins causes non-specific targeting to other tissues and low targeting to the nervous system. To overcome these drawbacks, long-circulation liposomes, specific active targeting liposomes, and other new types of liposomes have been developed over recent years (Gabizon et al., 2016; Li et al., 2019a).

Dopamine-PEGylated immunoliposomes (DA-PILs)—liposomes modified with polyethylene glycol and conjugated with antibodies—were developed as vehicles for dopamine in PD treatment. In a rat model of PD, the uptake of DA-PIL in the brains increased about 8-fold and 3-fold compared with that of DA and encapsulated DA-PEGylated liposomes (DA-PL), respectively (Kang et al., 2016). The physicochemical properties of liposomes can be modified by altering the phospholipids themselves or their ratio. Since dipalmitoyl phosphatidylcholine (DPPC) was the most pH-stable liposome found, with a sustained drug release at physiological pH (Yaroslavov et al., 2015), DPPC was selected as the carrier of curcumin to explore the therapeutic effect in human fetal astrocyte cell line SVGA model of neuroinflammation and reactive astrogliosis. Compared with free curcumin, LipoCur showed a significant downregulation of glial cell proliferation genes and a lower level of pro-inflammatory cytokines including IL-6, IL-1β, TGF-β, and TNF-α (Schmitt et al., 2020). In addition, Cyclosporine A (CsA) in liposomal formulation (Lipo-CsA) inhibits the inflammation response, including myeloperoxidase (MPO) activity and TNF-α levels, in the model of ischemia reperfusion injury (I/R) cerebral injuries (Partoazar et al., 2017). Therefore, liposomes serving as drug-delivery systems increase the BBB penetration of drugs to improve the anti-inflammatory effect.

#### Solid Lipid NPs

Manufactured from synthetic or natural lipids, solid lipid NPs (SLNs) have a lipidic core, which enables them to stay in solid state at room and body temperatures (Cupaioli et al., 2014). SLNs are less toxic than cationic liposomes and are generally recognized as safe in humans. Besides, they have been proved to be physiologically tolerated and have higher drug delivery efficiency compared to other types of lipid-based NPs (Banerjee and Pillai, 2019; Raza et al., 2019).

In LPS-induced BV-2 microglial cells, curcumin-loaded solid lipid nanoparticles (SLCN) dose-dependently inhibited the levels of nitric oxide (NO) and pro-inflammatory cytokines, such as TNF-α, IL-1β, and IL-6, and this was more effective than curcumin alone (Ganesan et al., 2019). Similarly, SLCN provides a superior effect in anti-Aβ, anti-inflammatory, and neuroprotective outcomes than traditional curcumin in one-year-old 5xFAD AD mouse (Maiti et al., 2018). In addition, sesamol-loaded SLNs were developed and found to significantly alleviate the oxidative stress in intracerebroventricular (ICV)-streptozotocin (STZ)-induced male Wistar rats, suggesting they provide a promising strategy to mitigate neuroinflammation and memory deficits (Sachdeva et al., 2015). SLNs are clearly useful delivery systems to alleviate neuroinflammation and neuronal dysfunction.

### Nanoemulsions

Nanoemulsions (NEs) are a colloidal dispersion consisting of two immiscible liquids stabilized by surfactants. A typical NE usually contains water, oil, and an emulsifier at appropriate ratios. NEs show some excellent properties including good biocompatibility, kinetical stability, cell transport by paracellular and transcellular pathways, and prevention of hydrolysis and enzymatic degradation of residues (Nirale et al., 2020). NEs can be administrated through nasal and ocular delivery in addition to the oral and intravenous administrations (Karami et al., 2019a; Karami et al., 2019b; Nirale et al., 2020).

Chitosan-coated rosmarinic acid nanoemulsions (RA CNE) have been shown to offer protection by inhibiting cellular death and repairing the astrocyte redox state in LPS-induced neuroinflammation and oxidative stress in astrocyte cells (Fachel et al., 2020a). Based on these *in vitro* results, researchers further illustrated the neuroprotective effects of RA CNE on the alleviation of neuroinflammation, oxidative stress, and memory deficit in Wistar rats (Fachel et al., 2020b). In LPS-induced rat neuroinflammation models, the brain uptake of siRNA delivered by cationic nanoemulsions was almost five times higher than non-encapsulated siRNA. More importantly, siRNA nanoemulsions significantly reduced the level of TNF-α, a signaling molecule which aggravates inflammation. Therefore, nanoemulsions encapsulated with TNF-α siRNA were suggested to be potential candidates in the treatment of neuroinflammation (Yadav et al., 2016). Ropinirole, a dopamine agonist as combination therapy with levodopa, is widely used in the treatment of PD. However, its efficiency was limited by its low bioavailability and short half-life. After modification, the transdermal delivery of ropinirole NE gel exhibited better drug absorption and less irritation and toxicity for the skin compared to ropinirole alone (Azeem et al., 2012).

### Polymer-Based NPs

#### Polymeric NPs

Polymeric NPs consist of amphiphilic block copolymers with varying hydrophobicities. They can be categorized into two groups: natural and synthetic polymeric NPs. Synthetic polymeric NPs can be manufactured via nanoprecipitation or the double emulsion method. Owning to the core–shell structure, polymeric NPs are able to encapsulate slow-release hydrophobic drugs and prolong circulation time. The surface of polymeric NPs can be decorated with ligands for targeted drug delivery. Therefore, polymeric NPs are considered drug carriers with high biological activity and bioavailability and have a high therapeutic index (Chen et al., 2015; Zielinska et al., 2020).

Natural polymeric macromolecules mainly include chitosan, alginates, dextrane, gelatin, collagen and their derivatives. Chitosan often derives from exoskeletons of crustaceans and cell walls of fungi and is a cationic polymer. As the second most abundant natural polysaccharide, chitosan, together with chitosan oligosaccharide and its derivatives, have been widely applied as the material of nano-carriers for the treatment of neuroinflammation. Besides, chitosan have neuroprotective effects in AD by inhibiting Aβ, acetylcholinesterase (AchE), oxidative stress, and neuroinflammation (Ouyang et al., 2017). Chitosan-coated synergistically engineered nanoemulsion of Ropinirole and nigella oil was suggested as a potential therapeutic strategy for PD by downregulating the NF-κB signaling pathway and inhibiting lipid peroxidation (Nehal et al., 2021). Alginate is an acidic polysaccharide from various marine brown algae. Alginate-derived oligosaccharide (AdO) was reported to significantly reduce the level of nitric oxide (NO) and prostaglandin E2 (PGE2), as well as the secretion of other proinflammatory cytokines. Furthermore, AdO significantly attenuated the overexpression of toll-like receptor 4 (TLR4) and NF-κB induced by LPS in BV2 cells (Zhou et al., 2015). In addition, alginate micro-encapsulation of mesenchymal stromal cells could modulate the neuroinflammatory response by decreasing the production of PGE2 in LPS induced astrocytes and microglia (Stucky et al., 2015; Stucky et al., 2017).

Synthetic polymers include polyesters and their copolymers, polyacrylates and polycaprolactones. Compared with natural molecules, their synthesis conditions can be controlled to regulate chain length, composition, and degradation to perform multiple functions (Colmenares and Kuna, 2017). In addition, synthetic polymers have been proved to possess relatively low toxicity profiles. Polymeric surface modification has been used to minimize the uptake by the reticuloendothelial system, thus increasing blood circulation half-life, which is a promising strategy to improve controlled drug release for long periods (Modi et al., 2010). Currently, poly-lactic-co-glycolic acid (PLGA), which is approved by United States Food and Drug Administration (FDA) for human application, is the most commonly studied polymer with good biocompatibility and biodegradability (Pavot et al., 2014; Younas et al., 2019). A novel brain-target nanoparticle, poly (lactide-co-glycolide)-block-poly (ethylene glycol) (PLGA-PEG) conjugated with B6 peptide and loaded with curcumin (PLGA-PEG-B6/Cur) was designed (Fan et al., 2018). Compared with native Cur, PLGA-PEG-B6/Cur significantly improved the spatial learning and memory ability of APP/PS1 mice by increasing the average half-life, decreasing metabolism, and maintaining the release of Cur, which showed potential for use in the treatment of AD. In addition, PEGylated-PLGA nanoparticles of epigallocatechin-3-gallate (EGCG) were developed to improve drug stability and increase the brain delivery in the treatment of temporal lobe epilepsy. Indeed, immunohistochemistry and neurotoxicity studies confirmed reduced neuronal death and neuroinflammation (Cano et al., 2018). NPs also showed a better effect on the reduction of the frequency and intensity of epileptic episodes than EGCG. In some other studies, PLGA NPs were synthesized to transfer superoxide dismutase (SOD) in cerebral ischemic reperfusion injury (IR) injury mouse models, and the results showed that PLGA NPs were effective in reducing apoptosis, inflammatory markers (TNF-a, IL-1β, and TGF-β), and infarct volume (Yun et al., 2013). In addition, Foxp3 plasmid-encapsulated PLGA NPs was found to significantly reduce microglial activity and decrease the generation of pro-inflammatory cytokines including TNF-α, I L-1β, IL-6, cyclooxygenase (COX)-2, and inducible nitric oxide synthase (iNOS) (Shin et al., 2019). The cl PGP-PEG-DGL/CAT-Aco system (cross-linked dendrigraft poly-l-lysine nanoparticles modified with Pro-Gly-Pro (PGP)peptide and catalase (CAT), a neuroprotective enzyme) was developed (Zhang et al., 2017). In this system, leukocytes serve as ‘Trojan horses’ and freight the CAT penetrate across the BBB more effectively. In the middle cerebral artery occlusion (MCAO) model, the cl PGP-PEG-DGL/CAT-Aco system significantly enhanced the delivery of catalase to ischemic subregions and reduced the volume of brain infarct. Therefore, the studies reviewed suggest the effectiveness, drug protection, and long cycle life of synthetic polymers.

#### Dendrimers

Dendrimers consist of a group of highly ordered macromolecules synthesized through repetitive chemical reactions from a core with a structure (Araujo et al., 2018; Dias et al., 2020). They were first discovered in 1985 and have been extensively studied. Through covalent bonds and ion interactions or adsorption, dendrimers deliver drugs, genes and proteins with molecules loaded inside or bound to their surface to bring them across the BBB (Chauhan, 2018; Sherje et al., 2018).

The advantages of dendrimers include the following: 1) controlled biodistribution and pharmacokinetics; 2) high structural and chemical homogeneity, which facilitates pharmacokinetic reproducibility; 3) the ability to associate with various compounds and/or ligands, improving their solubility and specificity; 4) and numerous surface groups of dendrimers contribute to multifunctionality and/or high drug loads (Lyu et al., 2020; Sandoval-Yanez and Castro Rodriguez, 2020; Yousefi et al., 2020). However, their higher cost of production is a limitation compared to linear polymers. Moreover, the toxicity of dendrimers was reported by some studies. A temporary increase of liver aspartate aminotransferase (AST) and alanine aminotransferase (ALT) levels was observed in a macaque model accepting anionic AzaBisPhosphonate groups (ABP dendrimer). Injections of G0-G3 amine-terminated PAMAM dendrimers in a mouse air pouch model caused a significant increase in leukocyte infiltration (Durocher and Girard, 2016).

**FIGURE 3:**
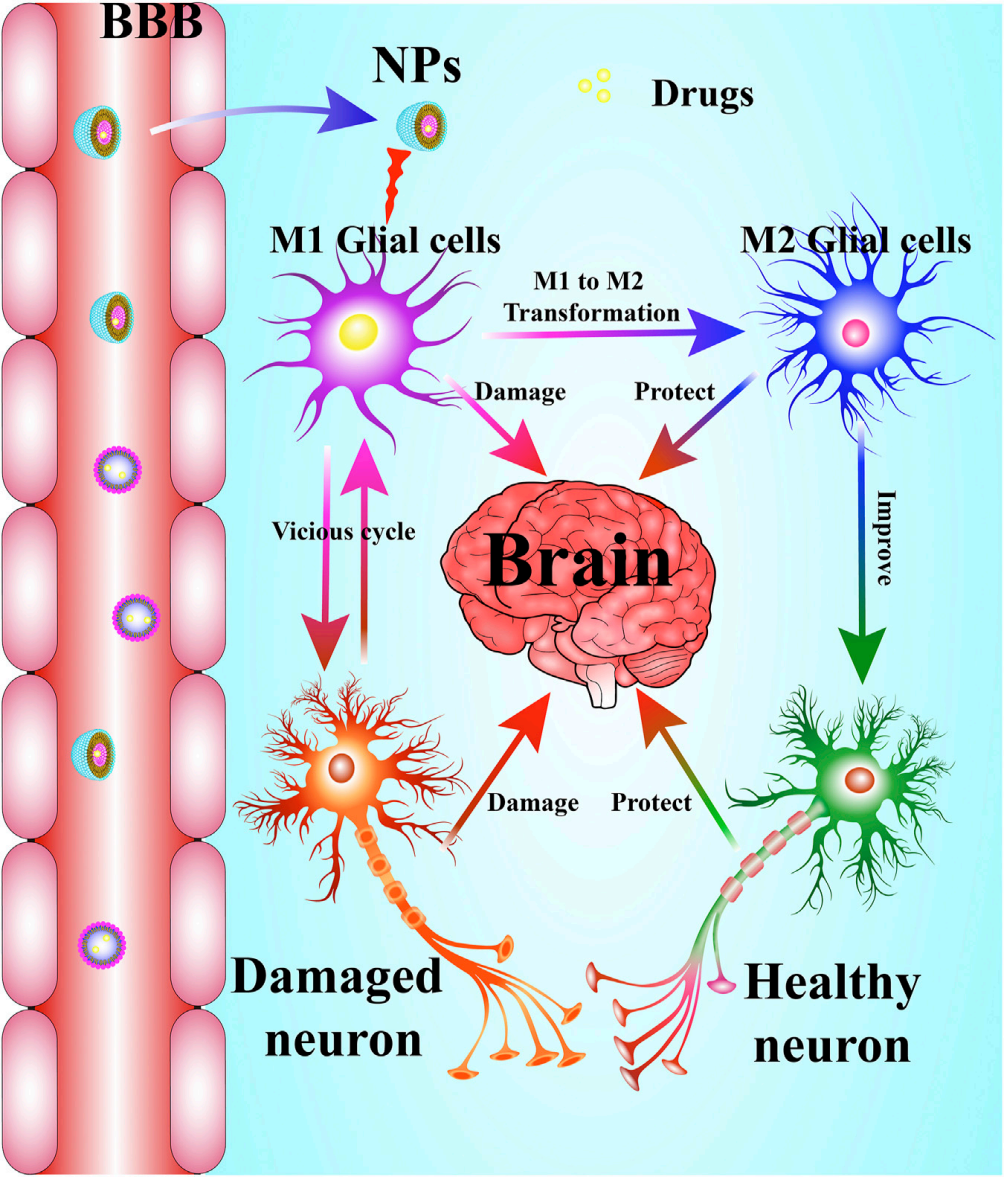
NPs serve as a drug delivery system in neuroinflammation-mediated CNS-related diseases. NPs delivery systems help drugs cross the BBB to inhibit over-activated microglia and its resultant neuroinflammatory response, which promotes the transformation of M1-type microglia into M2-type microglia and improves neuronal viability.

However, other researchers found that dendrimers were beneficial to human health. Dendrimer-based N-acetyl-l-cysteine (NAC) could be a therapy for neuroinflammation and cerebral palsy (CP) using a CP rabbit model induced by maternal intrauterine endotoxin by increasing the concentration of GSH in astrocytes and inhibiting neuroinflammation as indicated in GSH, 4-HNE, NT-3, 8-OHG, NF-kB, and TNF-a. Further study found that dendrimers are nontoxic, nonimmunogenic, and can be cleared completely through the kidneys (Kannan et al., 2012). Dendritic polyglycerol sulfates (dPGS) have been shown to be multivalent inhibitors of inflammation (Dernedde et al., 2010) and potent complement inhibitors (Silberreis et al., 2019). It was reported that dPGS interfered with Aβ fibril formation and reduced the production of the neuroinflammagen lipocalin-2 (LCN2) in astrocytes through its direct binding to Aβ_42_ and interaction with Aβ_42_. In addition, dPGS could normalize the impaired neuroglia cell and prevent the loss of dendritic spines at excitatory synapses in the hippocampus (Maysinger et al., 2018). Therefore, dPGS might be helpful in the treatment of neuroinflammation and neurotoxicity in AD and other neurodegenerative diseases. Moreover, fourth-generation poly amidoamine (PAMAM) dendrimers were synthesized by Li et al. (Li et al., 2012). Sino, a potent anti-inflammatory and antioxidant drug was combined with hydroxyl terminated generation-4 PAMAM dendrimer by Sharma et al. (2020b). D-Sino was demonstrated to be a potential therapy for attenuating inflammation in TBI at early stage through inhibiting the pro-inflammatory cytokines, including TNF-α, IL-1β, CCL-3, and IL-6, reducing the level of iNOS and NO, and inhibiting NF-κB activation and its nuclear translocation (Sharma et al., 2020b). Researchers also demonstrated that NAC, based on G4-OH PAMAM dendrimers (D-NAC), could increase intracellular GSH levels and prevent extracellular glutamate release and excitotoxicity in microglia and astrocytes, compared with NAC alone (Nance et al., 2017). Therefore, dendrimers, especially PAMAM, are considered a promising drug delivery system for CNS disease associated with neuroinflammation (Table 2; Figure 3).

**TABLE 2:** Dendrimers for the inhibition of neuroinflammation and their mechanisms within *in vitro* and *in vivo* models.

Dendrimers	Diameter (nm)	Biological model	Treatment	Dose	Mechanism and detected inflammatory cytokines	Toxicity	References
***In vitro***							
D-mino	∼8.4 nm	LPS-induced BV2 cells	24 h co-culture	Concentration range of 50–500 µM	NO, TNF-a	50–500 µM did not show cytotoxicity	Sharma et al. (2017)
PEGOL-60	Not Given	LPS-induced BV2 cells	24 h co-culture	500 μg/ml	TNF-a, IL-4, IL-6, IL-10, and iNOS	>1,000 μg/ml did not show cytotoxicity for 24 h	Sharma et al. (2020a)
dPGS	13.55 ± 0.14 nm	Primary neuroglia and organotypic hippocampal slice cultures exposed to Aβ-42 peptide	Pre-treated for 1 h	1 M	Interfered with Aβ fibril formation and downregulation of LCN2	Not Given	Maysinger et al. (2018)
D-Sino	4.9 nm	LPS-induced RAW 264.7 cells	8 h co-culture	50 µg/ml, 100 µg/ml and 300 µg/ml	NF-κB pathway; TNF-α, IL-1β, CCL-3, IL-6, iNOS, and NO	>300 µg/ml did not show cytotoxicity, 500 µg/ml decreased cell viability to 82.7 ± 7.4%	Sharma et al. (2020b)
PAMAM-(COOH)46-(NAC)18	Not Given	LPS-induced BV2 cells	Pre-treated for 3 h	0.5 mM 2 mM, and 8 mM	ROS, NO, and TNF-α	0.04–0.59 mM did not show cytotoxicity for 24 h	Wang et al. (2009)
PAMAM	∼4 nm	Brain slice culture model from newborn rabbits exposed by endotoxin	4 h co-culture	5 ng in 10 μL of DPBS solution	More rapid diffusion and ability to “find” the less mobile activated microglia, increasing microglial uptake	Not Given	Zhang et al. (2016)
***In vivo***							
ABP Dendrimer	Not Given	Mouse model of MOG35–55-induced autoimmune encephalomyelitis	Intravenous injection in different time in prophylactic and therapeutic groups	10 mg/kg	IFN-γ, IL-17, and IL-10	Did not induce immunosuppression or systemic toxicity in nonhuman primates	Hayder et al. (2015)
D-NAC	5.4 nm	A rabbit model of cerebral palsy induced by maternal intrauterine endotoxin	Intravenous injection to newborn	1 mg/kg, 10 mg/kg	NF-κB pathway; GSH and TNF-α	Nontoxic, nonimmunogenic, and are cleared intact through the kidneys	Kannan et al. (2012)
TPP-D-NAC	7.5 ± 0.2 nm	A rabbit model of TBI induced by surgery	Intravenous injection at 6 h post-injury	0.5 µg/ml, 5 µg/ml, and 50 µg/ml	Targeted delivery to mitochondria	Did not exhibit any reduction in cell viability at the doses tested	Sharma et al. (2018)
shCCL20-CCR6	100 nm	Mouse model of rTBI induced by surgery	Intranasal and intravenous administration after 3rd, 4th and 5th TBI	Not Given	IL-6 and CCL20	Low doses did not show cytotoxicity	Mayilsamy et al. (2020)

#### Nanogels Solid Lipid NPs

Aqueous-based liquids can be used as supporting media for polymer gels by physical/chemical intercrossing. Nanogels are three-dimensional hydrogel particles composed of hydrophilic or amphiphilic polymer chains. Based on their structure, nanogels can be divided into four groups: hollow, multi-layered, core cross-linked, and hairy nanogels (Soni et al., 2016; Li et al., 2017; Hajebi et al., 2019). Since nanogels have many advantages over other delivery materials including adjustable size, swelling, biocompatibility, hydrophilicity, ease of preparation, and stimulus responsiveness. Thus, they offer a promising prospect for drug, gene, or imaging agents transport. It was found that activin B-loaded hydrogels (ABLH) could provide lasting release of activin B for over five weeks in an MPTP-induced male C57BL/6J mice model of PD. Additionally, ABLH significantly increased the density of tyrosine hydroxylase (TH) positive nerve fibers and induced a noticeable reduction in neuroinflammatory responses, suggesting that ABLH may be a promising drug candidate for PD (Li et al., 2016). Self-assembling hydrogels possess superior characteristics without any structural modifications, as they are self-releasing, stable, soluble, injectable, stimuli responsive, and almost nontoxic. As a result, they are considered optimal therapeutic materials. Zheng et al. (2019) reported that rhein hydrogels—natural herbal drug hydrogels—enter the LPS-induced BV2 microglia and bind to TLR4 easily to inhibit the nuclear translocation of p65 in the NF-κB signaling pathway, thus reducing neuroinflammation with a sustained effect. Besides, it showed minimal cytotoxicity compared to rhein alone (Zheng et al., 2019). Therefore, nanogels have been developed in new ways, and their potential as a treatment for neuroinflammation needs to be explored further.

#### Polymeric Micelles

Micelles are colloidally made from amphiphilic block copolymers which aggregate in aqueous solutions and consist of a hydrophobic core and a hydrophilic surface (Zhang et al., 2012; Li et al., 2017). The mechanism of acute ischemic stroke includes oxidative stress, neuroinflammation, and cerebrovascular injury, which might lead to neuronal death. Lu et al. (2019) encapsulated rapamycin in self-assembled micelles consisting of ROS-responsive and fibrin-binding polymers. They found that the microthrombus-targeting micelles eliminated ROS generation and contributed to micelle polarized M2 microglia repair, thereby enhancing neuroprotection and blood perfusion.

## Inorganic NPs

### AuNPs

AuNPs are a type of inorganic nanoparticle which play an important role in pharmacology, sensing (Uehara, 2010), and bio-imaging (Kim et al., 2009; Duncan et al., 2010; Hutter and Maysinger, 2011; Yoo et al., 2017b) with a suitable size and shape. Although AuNPs are widely considered to be safe and have low phototoxicity (Li et al., 2019b), they still induce gold toxicity and the hepatobiliary elimination of AuNPs has attracted considerable attention (Bahamonde et al., 2018; Park et al., 2019). In neurodegenerative disease, AuNPs are reported to suppress the pro-inflammatory responses in a microglial cell line by inducing polarization toward the M2 phenotype, which is beneficial for CNS repair and regeneration (Xiao et al., 2020).

Emerging evidence showed that AuNPs regulated inflammatory signaling by inhibiting the TNF-α pathway and downregulating the NF-κB signaling pathway (Xiao et al., 2020). The mice injected intracerebroventricularly with streptozotocin (STZ) exhibited sporadic AD symptoms, activation of the NF-κB signaling pathway, and increased secretion of IL-1β, while the treatment of AuNPs significantly inhibited the pro-inflammatory response via the NF-κB pathway (Muller et al., 2017).

Furthermore, there are many AuNPs-modified drugs which are more effective as anti-inflammatories than AuNPs or drugs alone. IL-4 is an anti-inflammatory cytokine that can decrease pro-inflammatory cytokines (TNF-α and IL-6) and ameliorate the chronic inflammatory process (Casella et al., 2016). Compared to the AuNPs treatment alone group, the combination of okadaic acid and AuNPs significantly increased the level of IL-4 both in the hippocampus and cortex regions, suggesting that AuNPs together with okadaic acid exert a synergistic anti-inflammatory effect (Dos Santos Tramontin et al., 2020). In BV-2 cells, gold-quercetin NPs were demonstrated to have stronger anti-inflammatory effects than quercetin or AuNPs alone by decreasing the expression of inflammation-producing enzymes (COX-2 and iNOS) at both the transcriptional and translational levels (Ozdal et al., 2019). *Ephedra sinica* Stapf-AuNPs reduced pro-inflammatory cytokine levels and ROS production by downregulating the IKK-α/β, NF-κB, JAK/STA T, ERK-1/2, p38 MAPK, and JNK signaling pathways, upregulating the expression of HO-1 and NQO1, and by activating Nrf2 and AMPK in BV-2 microglial cells. In addition, the combination of AuNPs and n-acetylcysteine (NAC) significantly attenuated sepsis-induced neuroinflammation by decreasing myeloperoxidase activity and proinflammatory cytokines production, as compared with NAC or AuNPs treatment alone (Petronilho et al., 2020). Anthocyanins administered either alone or loaded with PEG-AuNPs reduced Aβ_1-42_-induced neuroinflammation and inhibited neuronal apoptosis by constraining the p-JNK/NF-κB/p-GSK3β pathway in BV2 cells and Aβ_1-42_-injected mice; anthocyanins loaded with PEG-AuNPs exhibited a stronger effect than anthocyanins alone (Kim et al., 2017). Moreover, l-DOPA-AuNF, a multi-branched nanoflower-like gold nanoparticles based on l-DOPA, efficiently improved the penetration of l-DOPA across the BBB (Gonzalez-Carter et al., 2019), which provides evidence for the further development of drugs with potent anti-inflammatory effects that cannot cross the BBB. Therefore, AuNPs, and especially AuNP-modified drugs, exhibit powerful anti-inflammatory effects against neurodegenerative disease (Table 3).

**TABLE 3:** AuNPs for neuroinflammation and their mechanisms as part of *in vitro* and *in vivo* models.

Cells/animals	Diameter (nm)	Treatment	Dose	Mechanism and detected inflammatory cytokines	References
***In vitro***					
BV2 cells	27 nm	24 h co-culture	100 µg/ml	iNOS and COX-2 mRNA	Ozdal et al. (2019)
BV2 cells	100 nm	24 h co-culture	20 µg/ml	NO, PGE2, IL-6, and IL-1β	Xue et al. (2019)
BV2 cells	35.04 ± 4.02 nm	24 h co-culture	>20 μg/ml	NF-κB, JAK/STAT, MAPK, and PLD pathways	Park et al. (2019)
				NO, PGE2, TNF-α, IL-1β, and IL-6	
BV2 cells, N2a cells	1.87 ± 0.14 nm	24 h co-culture	<5 µg/ml	NF-κB pathway	Xiao et al. (2020)
				IL-1β, IL-6, TNF-α, IL-10, and iNOS	
Mouse microglia N9 cell line	Not Given	24 h co-culture	10 µg/ml	NO	Gonzalez-Carter et al. (2019)
***In vivo***					
Wistar male rats	20 nm	The injection was given every 48 h over 21 days, beginning 24 h after AD model induction	2.5 mg/kg	IL-1β, IL-4, and TNF-α	Dos Santos Tramontin et al. (2020)
C57BL/6 mice	100 nm	C57BL/6 mice were induced with Parkinsonism for 5 consecutive days and treated only with 20 mg/kg body wt. of *Paeonia moutan*–AuNPs for 14 days	20 mg/kg	NO, PGE2, IL-6, IL-1β, and TNF-α	Xue et al. (2019)
C57BL/6 mice	1.87 ± 0.14 nm	The OGD-challenged brain slices were treated with AuNCs (0, 2 or 5 ug/mL, 0 ug/mL served as OGD controls). After 48 h treatment, the samples were fixed using 4% PFA.	5 μg/ml	NF-κB pathway	Xiao et al. (2020)
				IL-1β, IL-6, TNF-α, IL-10, iNOS, and ROS	
Male Wistar rats	20 nm	Rats received 50 mg/kg of AuNP and/or NAC (20 mg/kg) s.c. immediately after surgery and 12 h after surgery	50 mg/kg	TNF-α, IL-1β, and IL-6	Petronilho et al. (2020)
Wistar male rats	20 nm	The intraperitoneal GNPs treatment was initiated 48 h after administration of streptozotocin. GNPs administration frequency was every 48 h until the 21st after stereotactic surgery	2.5 mg/kg	NF-kB pathway	Muller et al. (2017)
				IL-1β	

### Iron Oxide Nanoparticles (IONPs)

IONPs belong to the ferrimagnetic class of magnetic materials, which are widely used in biomedical and bioengineering applications (Figuerola et al., 2010). Magnetic NPs have shown great promise in many fields (Dinali et al., 2017). Superparamagnetic iron oxide nanoparticles (SPIONPs) are applied in magnetic resonance imaging (MRI), magnetic particle imaging (MPI) and targeted drug delivery (Xu et al., 2011; Du et al., 2013; Khandhar et al., 2013; Jin et al., 2014; Schleich et al., 2015). SPIONPs have been extensively used for diagnosis to visualize tumors and metastases in liver (Choi et al., 2006), and for angiography as a blood pool agent to visualize inflammatory lesions such as atherosclerotic plaques (Neuwelt et al., 2015). In addition, IONPs also suppress the production of IL-1β in LPS-stimulated microglia (Wu et al., 2013). Therefore, IONPs are mainly employed to diagnose and suppress inflammatory lesions in neurodegenerative diseases.

### Silica Nanoparticles (SiO_2_NPs)

SiO_2_NPs, one of the most broadly exploited nanomaterials, have been utilized in a variety of industries (Vance et al., 2015). SiO_2_ NPs have been widely applied in the pharmaceutical industry to encapsulate water-insoluble therapeutic agents to improve their dispersal in aqueous media (Durfee et al., 2016; Echazu et al., 2016). Small-sized SiO_2_ has potential applications in the delivery of diagnostic and therapeutic agents across the BBB and brain imaging (Liu et al., 2014). Importantly, SiO_2_ exposure does not affect cell viability on different neural cells and does not induce neuroinflammation (Murali et al., 2015; Ducray et al., 2017). However, long-term NPs exposure leads to mood dysfunction and cognitive impairment and alters the synapse by activating MAPKs (You et al., 2018). Therefore, SiO_2_NPs can pass through the BBB, and their potential in the treatment of neuroinflammation needs to be explored.

### Nanocarbon Lipid-Based NPs

#### Carbon Nanotubes (CNTs)

CNTs are tubular structures made of a layer of graphene rolled into a cylinder (Eatemadi et al., 2014). These NPs are classified as single-walled carbon nanotubes (SWCNTs) or multi-walled carbon nanotubes (MWCNTs) according to the number of wall sheets in their structure (Salvador-Morales et al., 2006). The potential advantage of CNTs is their capacity to integrate with neurons and enhance neuronal functions as substrates for neuronal growth in different neuron cells (Hu et al., 2004; Matsumoto et al., 2007; Cellot et al., 2009).

After modification by polymers, CNTs can offer additional sites for conjugation of other molecules (Wong et al., 2013). There are some contradictions concerning the effect of CNTs on the nervous system. It was reported that SWCNTs exert an anti-inflammatory effect and protect neurons from ischemic damage in a rat stroke model (Nunes et al., 2012). The release of dexamethasone by polypyrrole/CNTs led to the attenuation of lipopolysaccharide (LPS)-induced microglia activation (Luo et al., 2011). Kermanizadeh et al. (2014) demonstrated downregulation of IL-1β after MWCNT exposure, indicating the inhibition of neuroinflammation. Meanwhile, others found an increase in the expression of IL-1β in mice following exposure to MWCNTs (Hamilton et al., 2013; Kido et al., 2014; HelmyAbdou et al., 2019). It has been reported that oxidation-shortened amino-functionalized MWNT and amino-functionalized MWNT induced a transient increase in almost all pro-inflammatory cytokines (Bardi et al., 2013). Rats exposed to MWCNTs showed an increase in the expression of IL-1β compared with a control group, while the rate of TNF-α expression in male albino rats was significantly increased after MWCNT exposure (Kido et al., 2014; HelmyAbdou et al., 2019). However, another study revealed a decrease in the rate of TNF-α expression after MWCNT exposure (Kermanizadeh et al., 2014). In addition, MWCNT exposure resulted in neuroinflammatory responses via BBB impairment in cerebrovascular endothelial cells treated with serum from MWCNT-exposed mice (Aragon et al., 2017). Therefore, most CNTs can be used as an effective drug delivery system for neuroinflammation.

#### Graphene Quantum Dots (GQDs)

GQDs exhibit similar physical and chemical properties to graphene. GQDs are novel 2D nanomaterials composed of graphene nanosheets with a lateral size below 10 nm and ten graphene layers forming the final particle (Ponomarenko et al., 2008; Zhou et al., 2012; Li et al., 2013). GQDs have been considered to alleviate immune-mediated neuroinflammation in a dark agouti (DA) rat model of chronic relapsing experimental autoimmune encephalomyelitis (EAE) via the activation of MAPK/Akt signaling and the suppression of the encephalitogenic Th1 immune response, as well as inflammatory cytokine IL-10, IL-17, and IFN-γ (Tosic et al., 2019). In addition, GQDs inhibit fibrillization of α-synuclein, trigger their disaggregation, and rescue neuronal death against PD (Kim et al., 2018b). GQDs have a potential therapeutic effect for AD by inhibiting the aggregation of Aβ_1-42_ peptides (Mahmoudi et al., 2012; Liu et al., 2015; Wang et al., 2018). Besides this, curcumin-GQDs can be used as a platform to sense APO e4 DNA, which is responsible for AD (Mars et al., 2018). Therefore, GQDs are considered a promising therapeutic strategy for neuroinflammation and neurodegenerative disease.

## Neurotoxicity of Nanomaterials

Nanomaterials have wide applications in neutral inflammation therapy with exciting therapeutic effects. However, some researchers have raised questions about the toxicity of nanoparticles, because at nanoscale many atoms may become very active. Therefore, toxicity, and especially neurotoxicity, of nanoparticles for neuroinflammation therapy must be taken into account.

Neurotoxicity refers to any adverse effect on the structure, function, or chemistry of the nervous system produced by physical or chemical causes (Teleanu et al., 2019). The main mechanisms for neurotoxicity involve the excessive production of reactive oxygen species leading to oxidative stress; the release of cytokines causing neuroinflammation; and dysregulations of apoptosis leading to neuronal death (Teleanu et al., 2018). Neurotoxicity of nanoparticles is closely connected with different parameters of nanoparticles like their shape, dosage, size, surface area, and so on. Among the parameters, the size and surface area are the key determinants of toxicity (Saifi et al., 2018). NPs that are commonly used have been studied for potential neurotoxic effects. For example, AuNPs might cause astrogliosis, which is defined as an increase in the number and size of astrocytes and cognition defects including attention and memory impairment. Astrogliosis is closely connected with hypoxia, ischemia, and seizures in brain diseases, and is commonly observed in AD patients (Saijo et al., 2010; Flora, 2017). A high dose of anatase TiO_2_NPs significantly increased the IL-6 level in plasma and brain, suggesting that oral intake of anatase TiO_2_NPs could induce neuroinflammation and neurotoxic effects (Grissa et al., 2016). IONPs exposure may affect synaptic transmission and nerve conduction (Kumari et al., 2012), causing immune cell infiltration and neural inflammation apoptosis (Wu et al., 2010), inducing oxidative damage in the striatum but not in the hippocampus (Kim et al., 2013). A study showed the drug-free liposomes induced neuropathologic changes, specifically neuroinflammation and necrosis (Yuan et al., 2015). Another study showed that the accumulation of Polysorbate 80-modified chitosan nanoparticles induced neuronal apoptosis, a slight inflammatory response and increased oxidative stress (Huo et al., 2012). Generally, inorganic NPs show more frequent and severe toxicity than organic nanoparticles (Mohammadpour et al., 2019). Most NPs exhibit anti-neuroinflammatory effects either alone or by carrying anti-inflammatory drugs; however, some NPs induce neuroinflammation.

## Conclusion and Future Perspectives

Neuroinflammation is an inflammatory response within the CNS that is marked by the activation of microglia and astrocytes and the production of pro-inflammatory cytokines. Neuroinflammation is the common mechanism behind CNS-related diseases including acute brain injury, stroke, and neurodegenerative diseases. Neurodegenerative diseases are characterized by gradual cognitive or memory impairment and movement disorder. They pose a severe threat to people’s health and lower their quality-of-life, especially affecting the elderly population. The treatment of neuroinflammation is faced with many difficulties owing to the poor BBB penetration of drugs. Nanomaterials, an emerging therapeutic tool, may help overcome this obstacle and improve the effect of drugs on anti-neuroinflammation. NPs are a promising delivery system that can combine with drugs by dissolution, adsorption, encapsulation or covalent bonding and be used in the treatment of CNS disorders. The superiorities of NPs enable them to reduce enzymatic degradation, clearance by endothelial cells, and peripheral side effects, while increasing targeting and bioavailability and helping overcome the obstacle of the BBB.

**FIGURE 4:**
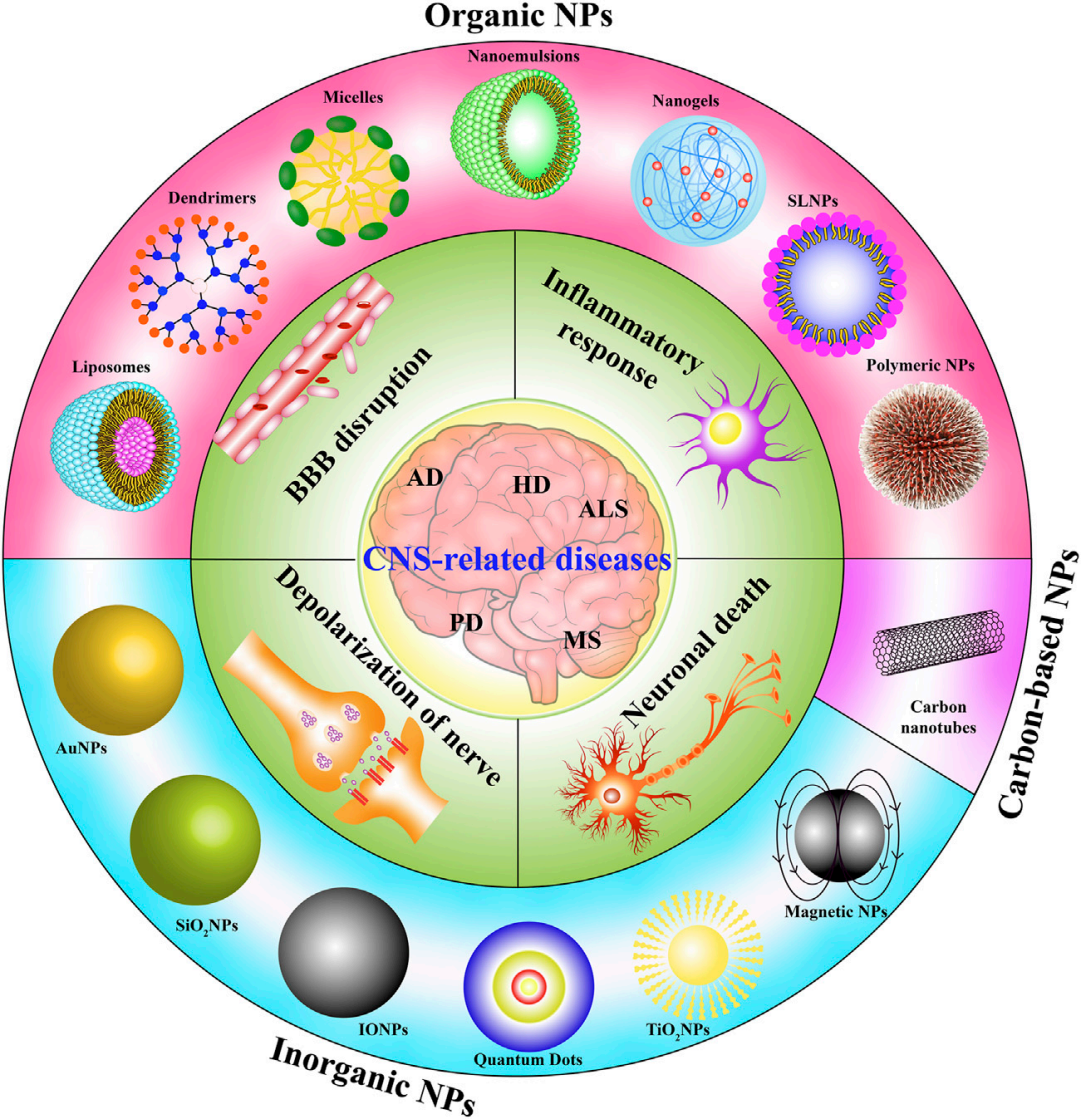
The potential therapeutic effect of NPs on the inflammatory response, neuronal death, depolarization of the nerve, and BBB disruption in CNS-diseases including AD, PD, HD, ALS, and MS.

To date, neurodegenerative diseases have affected millions of people worldwide, placing a serious financial and spiritual burden on societies and families. AD and PD are the most common neurodegenerative diseases. Although some drugs can alleviate their symptoms, there are still no drugs approved for the treatment of AD and PD. The BBB penetration of various drugs with potent neuroprotective effects, such as anti-inflammatory drugs in microglia and anti-apoptosis in neurons, is limited. The development of NPs represents a promising strategy for the improvement of the BBB penetration and neuroprotective effect of these drugs (Figure 4). For example, l-DOPA-AuNF improved the penetration of l-DOPA across the BBB (Gonzalez-Carter et al., 2019). ApoE3 polymeric nanoparticles loaded with donepezil showed an enhanced brain uptake of the drug, binding to amyloid beta with high affinity and accelerating its clearance (Krishna et al., 2019). Therefore, it is important to further develop the NPs with high BBB penetration capacity to encapsulate drugs with potent anti-inflammatory and anti-apoptosis effects such as galantamine, noferriamine, rivarasmine, risperidone, curcumin, quercetin, and ropinirole (Cao et al., 2016; Agrawal et al., 2018; Dudhipala and Gorre, 2020) for the treatment of AD and PD in the future.

In addition to the inherent characteristics of NPs, a variety of artificial designs have been developed to further improve performance by altering their size, surface area, surface charge, hydrophilicity and lipophilicity for the treatment of neuroinflammation. For example, binding with polyethylene glycol and polysaccharides prolongs the residence time of NPs. Transferrin-conjugated NPs exhibit higher permeability of the BBB. Prednisolone-loaded liposomes, nanoemulsions with oils rich in omega3 PUFA, polyclonal antibodies against brain-specific antigen and insulin-attached micelles, apolipoprotein E attached SLNs, G4HisMal, and D-mino dendrimers have all exhibited increased targeting of brain tissue (Naqvi et al., 2020).

The synthesis of multifunctional NPs is a hot research topic. Each NP has its own merits and drawbacks. In some cases, the properties of NPs are not compatible with drug binding, drug delivery, crossing the BBB, localization, and drug release (Kim et al., 2018a; Habibi et al., 2020). Therefore, by integrating NPs of different sizes, structures, and functions, multicomponent and multifunctional NPs are designed and their superior characteristics, (e.g. specific-targeting and long-circulation time) can be maximized. In recent years, some researchers have reported the application of PEG-cationic bovine serum albumin (Liu et al., 2013a), PEG-PLA NPs (Liu et al., 2013b), PEG–PLGA NPs (Zhang et al., 2014), chitosan-coated nanoemulsions (Fachel et al., 2020a), mSPAM (Rajendrakumar et al., 2018), and CeNC/IONC/MSN-T807 (Chen et al., 2018) as therapeutic strategies for neuroinflammation and neurodegenerative disease. Multifunctional NPs have extensive application prospects and warrant further exploration.

However, the drawbacks of NPs cannot be ignored. In the last decade, many studies reported that nanomaterials induce pro-inflammatory responses, apoptosis, and excessive oxidative stress of neurons in the brain. In addition, NPs were also demonstrated to accumulate in the liver, kidney and spleen, which may pose a threat to long-term health after administration. Considering these issues, the application of only those organic and degradable NPs with relatively minimal toxicity could be a possible solution. Furthermore, investigations of these nanomaterials in pharmacodynamics and pharmacokinetics are still limited, and their side effects remain to be explored.

NPs are still making their way from bench to clinical application, and many more studies are needed to solve the outstanding problems regarding the treatment of neuroinflammation.
